# Using adaptive neuro‐fuzzy inference system and multiple linear regression to estimate orange taste

**DOI:** 10.1002/fsn3.1149

**Published:** 2019-08-30

**Authors:** Marzieh Mokarram, Hosein Amin, Mohammad R. Khosravi

**Affiliations:** ^1^ Department of Range and Watershed Management, College of Agriculture and Natural Resources of Darab Shiraz University Shiraz Iran; ^2^ Department of Plant Production, College of Agriculture and Natural Resources of Darab Shiraz University Shiraz Iran; ^3^ Department of Electrical and Electronic Engineering Shiraz University of Technology Shiraz Iran

**Keywords:** adaptive neuro‐fuzzy inference system, Darab, multilayer perceptron, orange taste, vitamin C

## Abstract

In this research, some characteristic qualities of orange fruits such as vitamin C and acid content; weight; fruit and skin diameter; and red (R), green (G), and blue (B) values of the RGB color model for 70 samples were used to predict the taste of orange grown in Darab, southeast of Fars Province, Iran, by multiple linear regression (MLR) and adaptive neuro‐fuzzy inference system (ANFIS). To use MLR, firstly the most important input data were selected, and then, the best model to predict the taste of orange was applied. In this research, methodology of ANFIS consisted of selection of dependent orange taste, fuzzification, fuzzy inference rule, membership function, and defuzzification process. The predictive capability of these models was evaluated by various descriptive statistical indicators such as mean square error (*MSE*) and determination coefficient (*R* ^2^). The results showed that the prediction performance of the MLR model has a strong significant relationship between orange taste and vitamin C (0.897^**^), red color (0.901^**^), and blue color (0.713^*^). Also, the results of ANFIS model showed that with low error for train and check data increased the most accuracy for prediction of orange taste. Moreover, the results indicated that the success rate of taste determination for orange is higher by using ANFIS compared to the MLR. This research was to provide valuable information for orange taste.

## 1 INTRODUCTION

Artificial neural networks (ANNs) have been used to forecast soil, water, and vegetation characteristics (Ferreira, Callou, Josua, Tutsch, & Maciel, 2019; Liu, Yang, Ge, & Miao, 2006; Rad, Fanaei, & Rad, 2015). The ANN method does not need any specific function to model the relationship between inputs and outputs. However, a training procedure is used in the ANN method to link the input and output data (Schaap & Leij, 1998).

Usually in several years, ANNs because of their nonlinear characteristics have been applied (Yilmaz & Kaynar, 2011). Different efforts have been conducted in relation to modeling different fruit parameters by ANN methods. Simões, Costa, Hirakawa, and Saraiva (2002) used artificial neural network using RGB color systems for orange sorting. Kondo, Ahmad, Monta, and Murase (2000) forecasted the sugar content or pH of orange fruit using ANN.

Scala et al. (2013) used an ANN model for forecasting quality properties of fruits during convective dehydration. Choi, Kwon, Bae, and Kim (2016) used DNN (deep neural network) to predict fruit characteristics. The results showed that the model to perform the detection of seven fruits was suitable.

In order to forecast sugar content and taste of orange, Kondo et al. (2000) used image processing and ANNs. Also, the determination of orange taste using image processing and ANFIS (Fuzzy Inference Systems) was studied by Adelkhani, Beheshti, Minaei, Javadikia, and Ghasemi‐Varnamkhasti (2013). The results showed that the accuracy of model for forecasting orange taste was 93%.

Moreover, the studies showed that using spectral reflectance could predict characteristic fruits. So that, the visible light of the spectrum reflectance to predict characteristics of trees (Valencia orange) such as fruit and leaf was used (Gausman, 2009).

In fact, according to fruit color and pigment content, spectral reflectance can predict the quality of fruits (Li et al., 2018). Fruit color is applied as a qualitative as well as quantitative character distinguishing the fruit quality (Kondo et al., 2007; Sun & Li, 2017; Tarantino, Lops, Disciglio, & Lopriore, 2018; Veerappan et al., 2016; Zheng, An, Feng, & Wang, 2017).

The main aim of this work is to utilize the ANFIS method to predict orange taste. This method is based on Sugeno‐type system for the simulation and analysis of the mapping relationship between the vitamin C, acid, weight, fruit and skin diameter, red (R), green (G), and blue (B) as input data and orange taste as output data through the back‐learning multiplication process.

**Figure 1:**
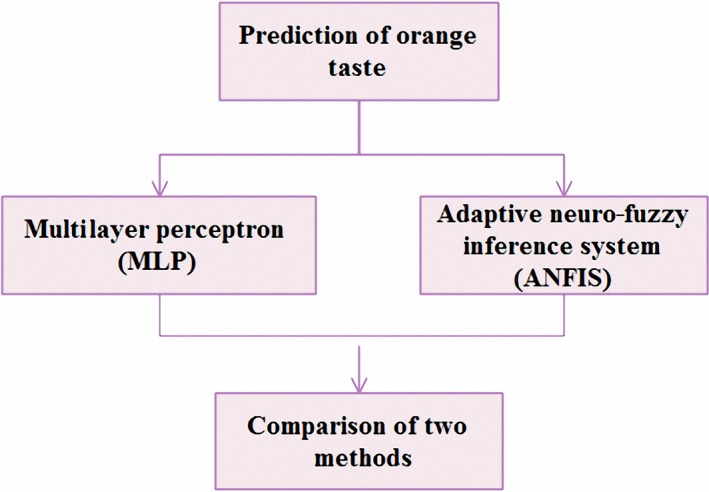
Flowchart for prediction of orange taste using ANFIS and MLR methods

So the aim of this research is employing the MLR and ANN modeling techniques such as ANFIS to determine the orange taste in Darab, southeast of Fars Province, Iran (Figure 1).

## 2 MATERIALS AND METHODS

### 2.1 Data

In order to predict orange taste, these parameters (acid, fruit weight, vitamin C, fruit diameter, skin diameter, red, green, and blue values of the RGB color) from 70 samples in different months were measured in Darab gardens, Fars Province, Iran. The summaries of them are shown in Table 1. For measurement of vitamin C and acid was used titration method. For weight were applied GF‐3000 model digital scales. Fruit diameter and skin diameter were measured by S‐R 400 model digital coliseum. Finally, by using MATLAB software, orange images were converted to a matrix.

**Table 1:** Summaries of input data for prediction of orange taste

Parameters	Vitamin C (mg/100)	Acid (mg/l)	Weight (g)	Fruit diameter (mm)	Skin diameter (mm)	Color		
						Red	Green	Blue
Minimum	48.00	0.33	22.70	62.06	2.40	71.50	69.50	8.00
Maximum	163.50	1.04	378.46	87.97	6.91	242.00	171.00	71.00
Average	109.27	0.56	234.54	74.39	4.38	158.11	117.73	24.91
STDEV	22.94	0.17	56.64	5.86	1.15	44.31	24.80	11.86

### 2.2 Multiple regression models

The general aim of multiple regressions is to determine the relationship between independent (vitamin C, acid, weight, fruit and skin diameter, red [R], green [G], and blue [B]) and dependent (orange taste) parameters for the investigation of designated goal. The regression equations were computed based on Equation 1:

(1) $$M=S_{0}+S_{1}X_{1}+S_{2}X_{2}+\cdots +b_{n}X_{n}$$

where *M* is the dependent variable, *S*_0_ is the intercept, *S*_1_ … *bn* are regression coefficients, and *X*_1_–*X_n_* are independent factors referring to basic orange characteristics.

### 2.3 Adaptive neuro‐fuzzy inference system (ANFIS)

Artificial neural networks are used as modeling tool to determine the best model between input and output variables. ANN models were used by several authors, for example, Alp and Cigizoglu (2007), Azmathullah et al. (2009), Bateni, Borghei, and Jeng (2007), Lee, Jeng, Zhang, and Hong (2007), Vali, Ramesht, and Mokarram (2013) and Mokarram and Bijanzadeh (2016).

**Figure 2:**
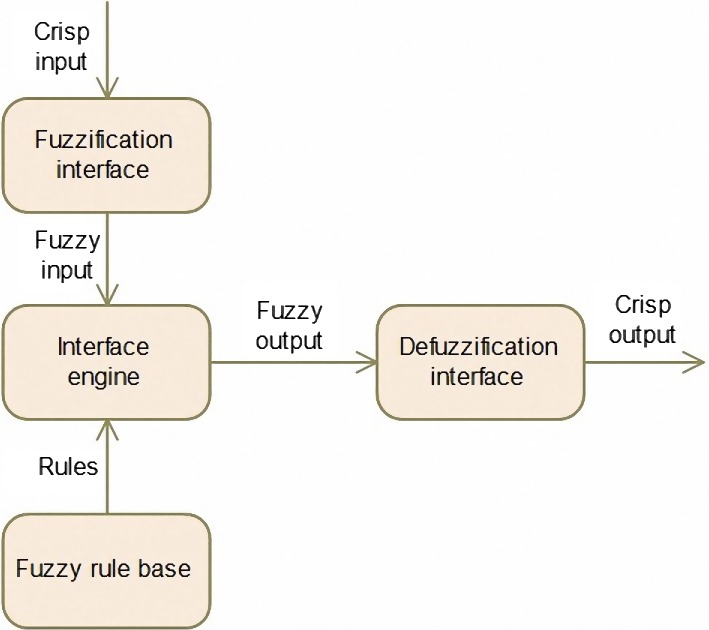
General architecture of the fuzzy inference system

The ANFIS is one of the ANN models that is a combination of fuzzy systems and ANN. The stage of ANFIS method is shown in Figure 2.

To forecast fuzzy rules, for eight inputs, a typical rule set with eight fuzzy rules and eight membership functions (MF) can be expressed as follows (Bui, Bui, Zou, Van Doan, & Revhaug, 2017):

(2) $$\begin{array}{c} \text{Rule}\;1:\;\text{If}\;x_{1}\;\text{is}\;T_{1},\;x_{2}\;\text{is}\;T_{2},\;x_{3}\;\text{is}\;T_{3},\;x_{4}\;\text{is}\;T_{4},\;x_{5}\;\text{is}\;T_{5},\;x_{6}\;\text{is}\;T_{6},\;x_{7}\;\text{is}\;T_{7},\;x_{8}\;\text{is}\;T_{8},\\ \text{then}\;f_{1}=k_{1}x_{1}+l_{1}x_{2}+m_{1}x_{3}+n_{1}x_{4}+o_{1}x_{5}+p_{1}x_{6}+q_{1}x_{7}+r_{1}x_{8}+r_{1}\\ \text{Rule}\;2:\;\text{If}\;x_{1}\;\text{is}\;T_{11},\;x_{2}\;\text{is}\;T_{22},\;x_{3}\;\text{is}\;T_{33},\;x_{4}\;\text{is}\;T_{44},\;x_{5}\;\text{is}\;T_{55},\;x_{6}\;\text{is}\;T_{66},\;x_{7}\;\text{is}\;T_{77},\;x_{8}\;\text{is}\;T_{88},\;x_{9}\;\text{is}\;T_{99},\\ \text{then}\;f_{2}=k_{2}x_{1}+l_{2}x_{2}+m_{2}x_{3}+n_{2}x_{4}+o_{2}x_{5}+k_{2}x_{6}+l_{2}x_{7}+m_{2}x_{8}+r_{2} \end{array}$$

where *x*_1_, *x*_2_, … *x_n_* are inputs; *f_j_* (*j* = 1 n) are output.

**Figure 3:**
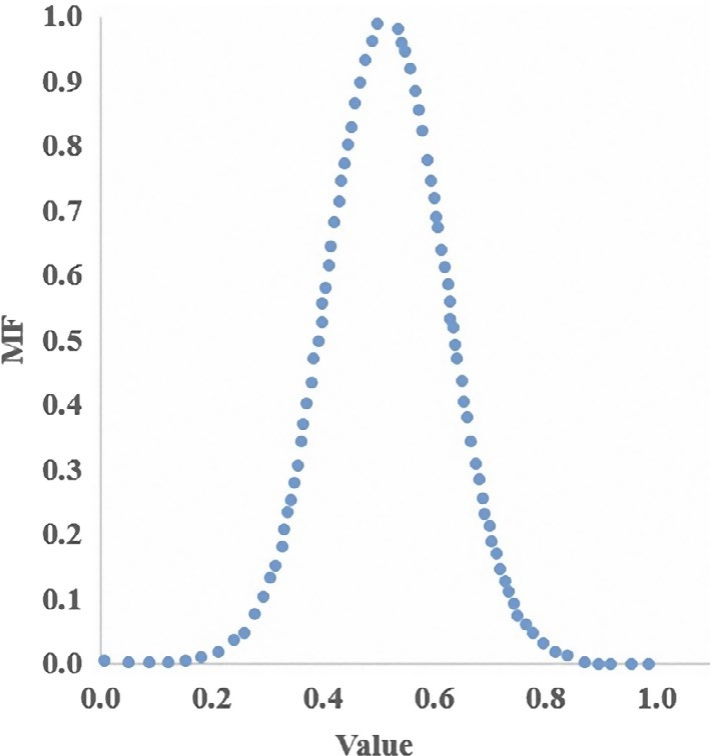
The membership function

For definition, membership function was used as Gaussian function. The Gaussian function is distinguished using the central value *m* and *a* standard deviation *k* more than 0. The membership function is shown in the following:

(3) $$\mu_{A}\left(x\right)=e^{-\frac{{\left(x-m\right)}^{2}}{2k^{2}}}$$

where *m* and *k* are arbitrary real constants. The membership function of Gaussian function shows that in Figure 3.

Membership function for eight input data and the rules are shown in the following:

(4) $$\begin{array}{c} w1=\mu_{1}x_{1}+\mu_{1}x_{2}+\mu_{1}x_{3}+\mu_{1}x_{4}+\mu_{1}x_{5}+\mu_{1}x_{6}+\mu_{1}x_{7}+\mu_{1}x_{8}+r_{1}\\ w2=\mu_{2}x_{1}+\mu_{2}x_{2}+\mu_{2}x_{3}+\mu_{2}x_{4}+\mu_{2}x_{5}+\mu_{2}x_{6}+\mu_{2}x_{7}+\mu_{2}x_{8}+r_{2}\\ w3=\mu_{3}x_{1}+\mu_{3}x_{2}+\mu_{3}x_{3}+\mu_{3}x_{4}+\mu_{3}x_{5}+\mu_{3}x_{6}+\mu_{3}x_{7}+\mu_{3}x_{8}+r_{3} \end{array}$$

The *j*‐th node of this layer computes the normalized firing strengths as (N):

(5) $$\begin{array}{c} {\bar{W}}_{j}=\frac{w_{j}}{\sum_{i=1}^{8}w_{j}}j=1÷8\\ {\bar{W}}_{j}f_{j}=\bar{w}\left(k_{j}x_{1}+l_{j}x_{2}+m_{j}x_{3}+n_{j}x_{4}+o_{j}x_{5}+p_{j}x_{6}+q_{j}x_{7}+r_{j}x_{8}\right)j=1÷8 \end{array}$$

The normalized firing strength (N) is computed in *j*‐th node of this layer. Moreover, the overall output (*µ*) obtained by ANFIS method is calculated in this layer.

(6) $$Q=\sum_{i=1}^{8}{\bar{w}}_{i}f_{i}=f$$

In total, ANNs consist of computing the outputs, compare the outputs with the desired target values, adjust the weights, and repeat the process.

One of the most widely used algorithms in the field of orange taste properties is the basic backpropagation, FCM, and hybrid algorithms. These algorithms minimize the difference between obtained outputs and desired targets by calculating some factors such as *MSE*, *RMSE*, and *MAE* factors.

#### 2.3.1 Network design

The ANFIS used in the study contains an eight‐layer feedforward neural network and implements TS (Takagi Sugeno) fuzzy inference system for a systematic method to making fuzzy rules from a given input–output dataset. The *RMSE* (root mean square error), *MAE* (mean absolute error), and correlation coefficient (*R*) were computed to provide an indication of goodness of fit between the observed and forecasted values.

### 2.4 Performance evaluation criteria

For determination of the precision of the forecasting capacity of the models, mean square error (*MSE*) and the coefficient (*R* ^2^) were used that can be calculated using Equations 7 and 8:

(7) $$R^{2}=1-\frac{\sum_{i=1}^{N}\left(y_{i}-{\hat{y}}_{i}\right)}{\sum_{i=1}^{N}{\left(y_{i}-{\bar{y}}_{i}\right)}^{2}}$$

(8) $$MSE=\frac{1}{T}\sum_{i=1}^{N}{\left(y_{i}-{\hat{y}}_{i}\right)}^{2}$$

In Equations 7 and 8, *T* depicts the number of data, *y_i_* is the desired output, and ${\hat{y}}_{i}$ is the predicted output.

## 3 RESULTS AND DISCUSSION

### 3.1 Orange analysis

**Figure 4:**
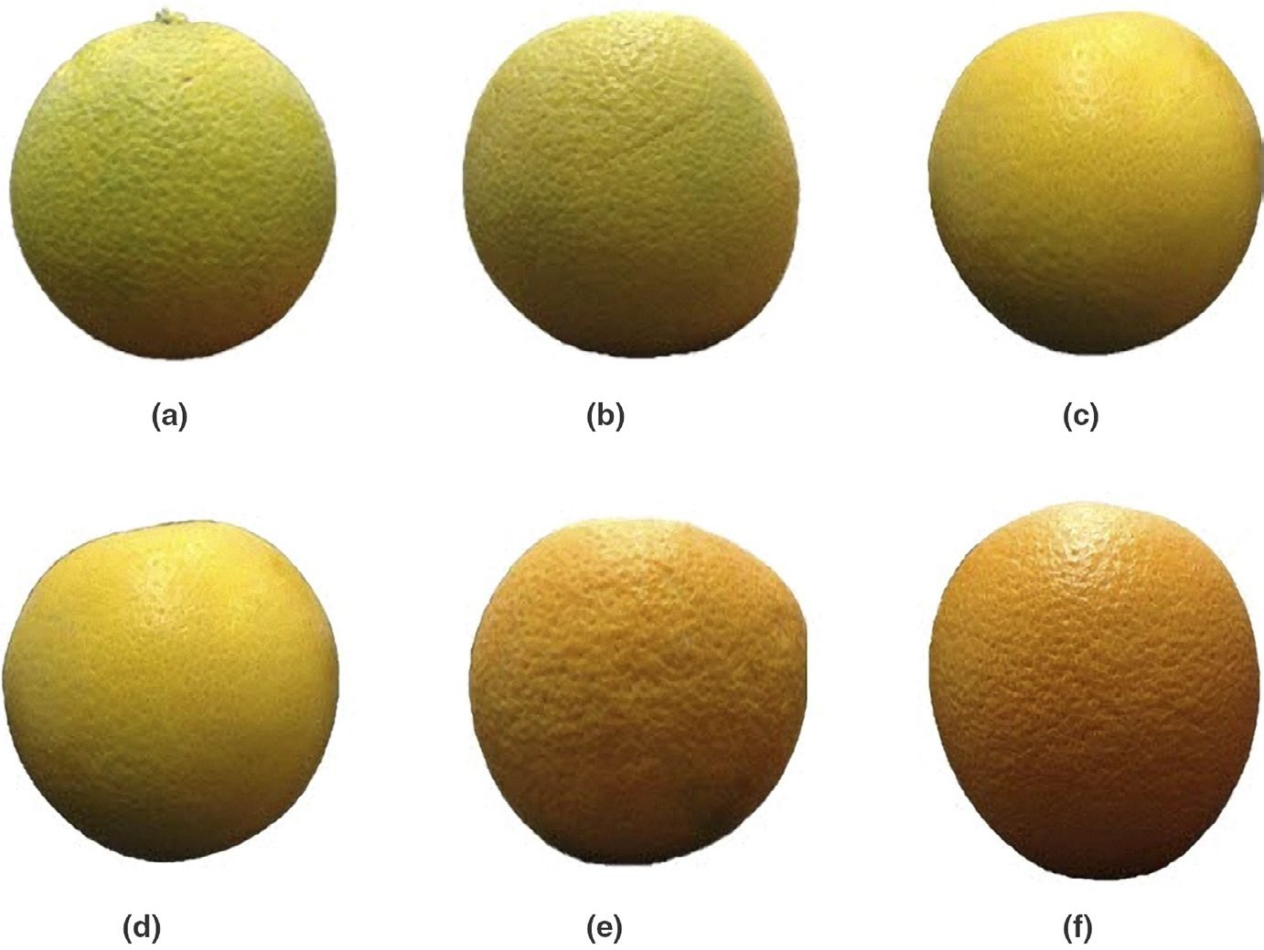
The samples orange in different months. (a): Early November; (b): Late November; (c): Early January; (d): Late January; (e): Middle February; (f): Middle March

In order to predict orange taste, 70 samples in different months were used (Figure 4). In addition, 70% of the whole data were used for training procedure, while it is 30% to test the obtained results (Tables 2 and 3).

**Table 2:** Descriptive statistics of the training dataset

Parameters	Vitamin C (mg/100 ml)	Acid (mg.m/L)	Weight (g)	Fruit diameter (mm)	Skin diameter (mm)	Color		
						Red	Green	Blue
Minimum	39	0.27	22.7	62.56	2.46	68	71	8
Maximum	153	0.88	378.46	87.97	6.81	242	170	91
Average	107.488	0.5304	239.816	74.1804	4.1474	148.96	119.82	25.38
STDEV	23.03	0.14	55.41	5.71	1.15	47.29	24.08	13.37

**Table 3:** Descriptive statistics of the testing dataset

Parameters	Vitamin C (mg.100 m/L)	Acid (mg.m/L)	Weight (g)	Fruit diameter (mm)	Skin diameter (mm)	Color		
						Red	Green	Blue
Minimum	57	0.38	22.7	61.56	2.34	75	68	8
Maximum	174	1.19	378.46	87.97	7	242	172	51
Average	111.06	0.59	229.25	74.59	4.61	167.26	115.64	24.44
STDEV	22.83	0.19	57.87	5.99	1.13	41.32	25.52	10.34

#### 3.1.1 Relationships between orange variables

The calculated *R* between orange taste and independent variables was investigated by means of SPSS V.22 software that are shown in Table 4. It was found that there was a positive and highly significant correlation between taste and vitamin C (0.897^**^), red color (0.901^**^), and blue color (0.713^*^) content.

**Table 4:** Simple linear coefficient correlations (*r*) among orange variables

Parameters	Taste	Vitamin C (mg.100 m/L)	Acid (mg.100 m/L)	Weight (g)	Fruit diameter (mm)	Skin diameter (mm)	Color		
							Red	Green	Blue
Taste	1	0.897*	0.001	−0.234	−0.319	0.256	0.901*	−0.183	0.713**
Vitamin C (mg.100 m/L)	0.897*	1	−0.074	−0.198	−0.349	−0.084	0.848*	−0.378	0.424
Acid (mg.100 m/L)	0.001	−0.074	1	−0.090	−0.043	0.216	−0.045	−0.249	−0.347
Weight (g)	−0.234	−0.198	−0.090	1	0.979*	0.169	−0.072	−0.050	−0.075
Fruit diameter (mm)	−0.319	−0.349	−0.043	0.979*	1	0.244	−0.178	0.073	−0.065
Skin diameter (mm)	0.256	−0.084	0.216	0.169	0.244	1	0.066	0.420	0.376
Red	0.901*	0.848*	−0.045	−0.072	−0.178	0.066	1	−0.419	0.705**
Green	−0.183	−0.378	−0.249	−0.050	0.073	0.420	−0.419	1	0.103
Blue	0.713**	0.424	−0.347	−0.075	−0.065	0.376	0.705**	0.103	1

* Correlation is significant at the .01 level (2‐tailed).

** Correlation is significant at the .05 level (2‐tailed).

### 3.2 Prediction of orange taste by using MLR

For predicting orange taste by the MLR model, the first most important input data were selected using the stepwise method, and then, linear interaction term of these basic orange characteristics was defined in the SPSS V.22 software. The results based on *R* ^2^ showed that model 1 with red color parameter was the best model for prediction of orange taste (Tables 5 and 6).

**Table 5:** MLR model summary for orange taste prediction

Model	*R*	*R* ^2^	Adjusted *R* ^2^	*SE* of the estimate	Change statistics				
					*R* ^2^ change	*F* change	*df*1	*df*2	Sig. *F* change
1	.901^a^	.811	.784	0.9847	.811	30.117	1	7	0.001

a Predictors: (Constant), Red.

**Table 6:** Performance indices (*R*, *R* ^2^, and *MSE*) and coefficients of variables for different MLR models for prediction of orange taste

aModel	Unstandardized coefficients		Standardized coefficients	*t*	Sig.	Correlations			Collinearity statistics	
	*B*	*SE*	*β*			Zero‐order	Partial	Part	Tolerance	VIF
1 (Constant)	−25.989	7.515		−3.458	0.011					
*R*	.211	.038	.901	5.488	.001	.901	.901	.901	1.000	1.000

a Dependent variable: taste.

**Figure 5:**
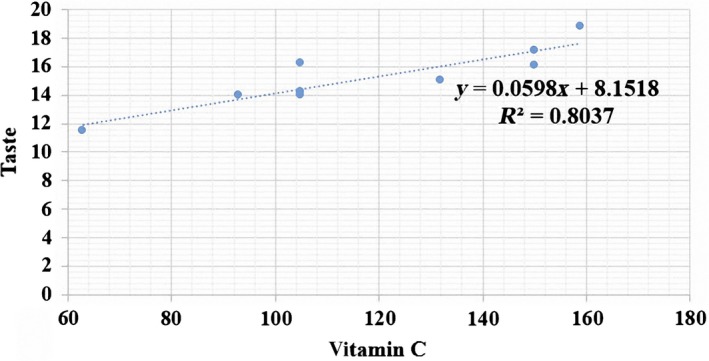
The scatter plot of the measured versus predicted orange taste using MLR

As it is obvious from Table 5, the *R* ^2^ value was .901 that proves the performance of the MLR method. In addition, Figure 5 shows the relationship between “taste” and “vitamin C” factors as a scatter plot in MLR method.

### 3.3 Prediction of orange taste using ANFIS

**Figure 6:**
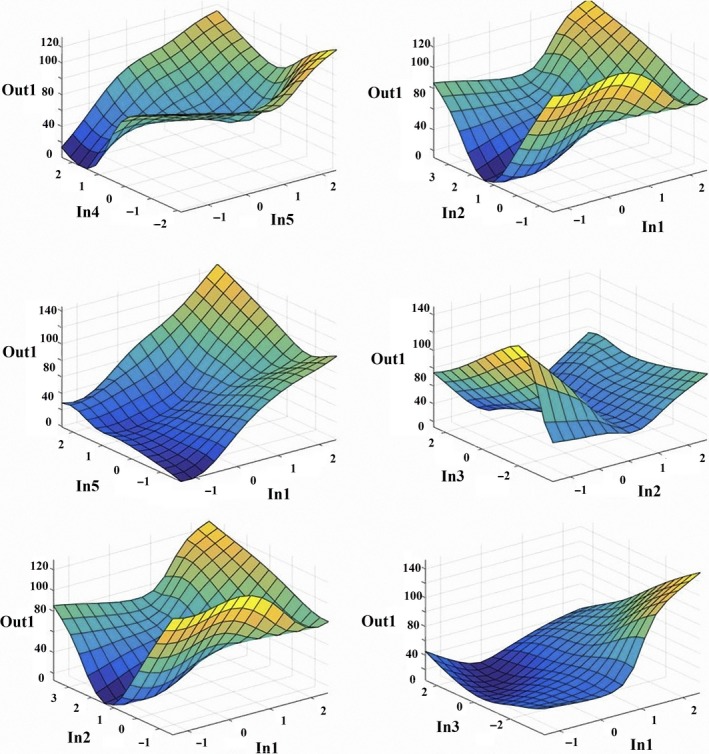
Relationship between one of the inputs and orange taste

The adaptive neuro‐fuzzy inference system (ANFIS) has been made using Fuzzy Logic Toolbox graphical user interface (GUI) tools in MATLAB R2014a. Input data for prediction of orange taste were vitamin C, acid, weight, fruit diameter, skin diameter, red, green, and blue wave. The Surface Viewer was used for presenting the mapping from nine inputs to one output for prediction of orange taste (Figure 6).

**Figure 7:**
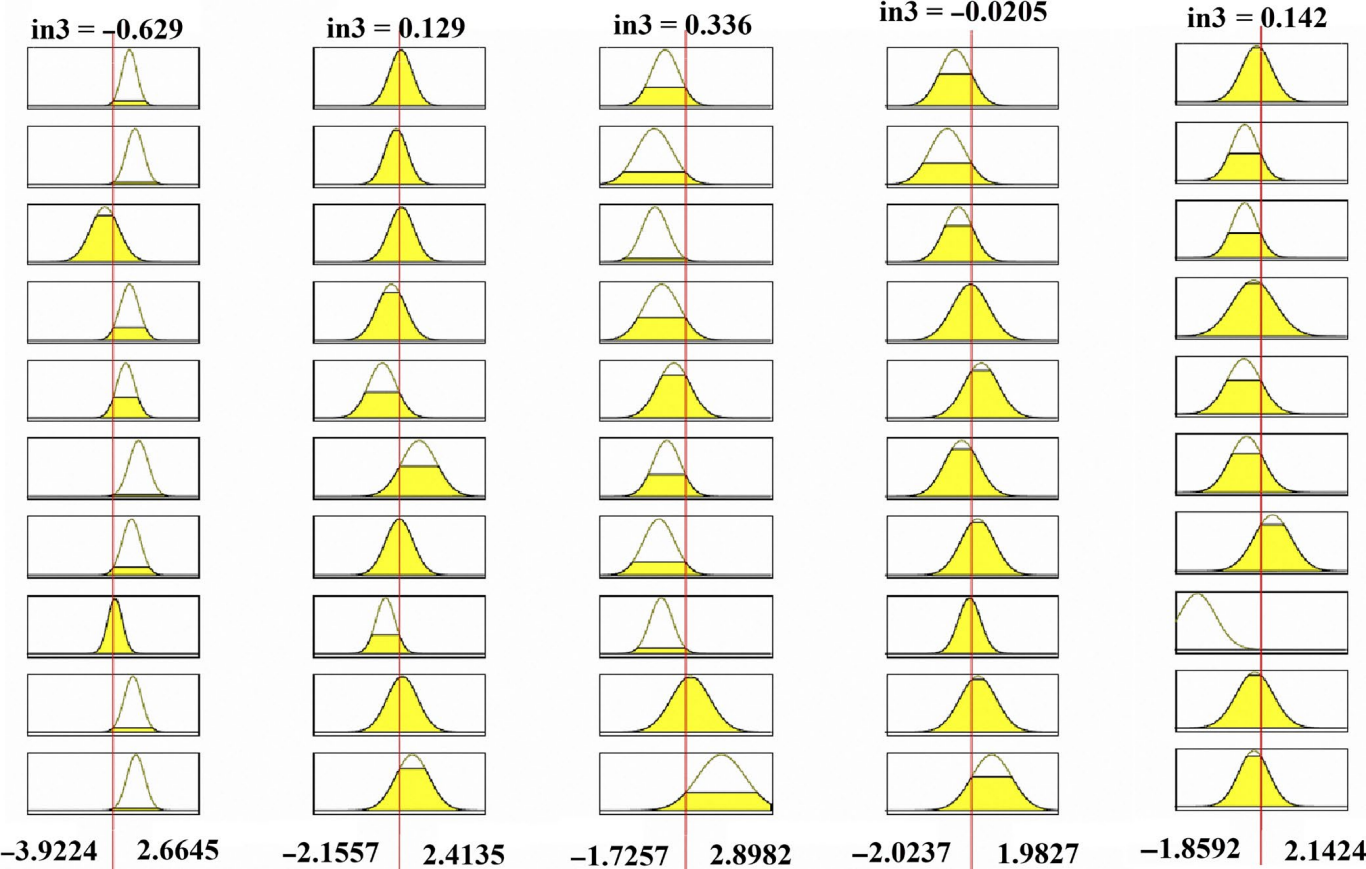
Inference system design

The Rule Viewer presents a sort of micro view of the fuzzy inference system as shown in Figure 7. Nine input values were selected by feature section algorithm.

### 3.4 Validation of the results

**Figure 8:**
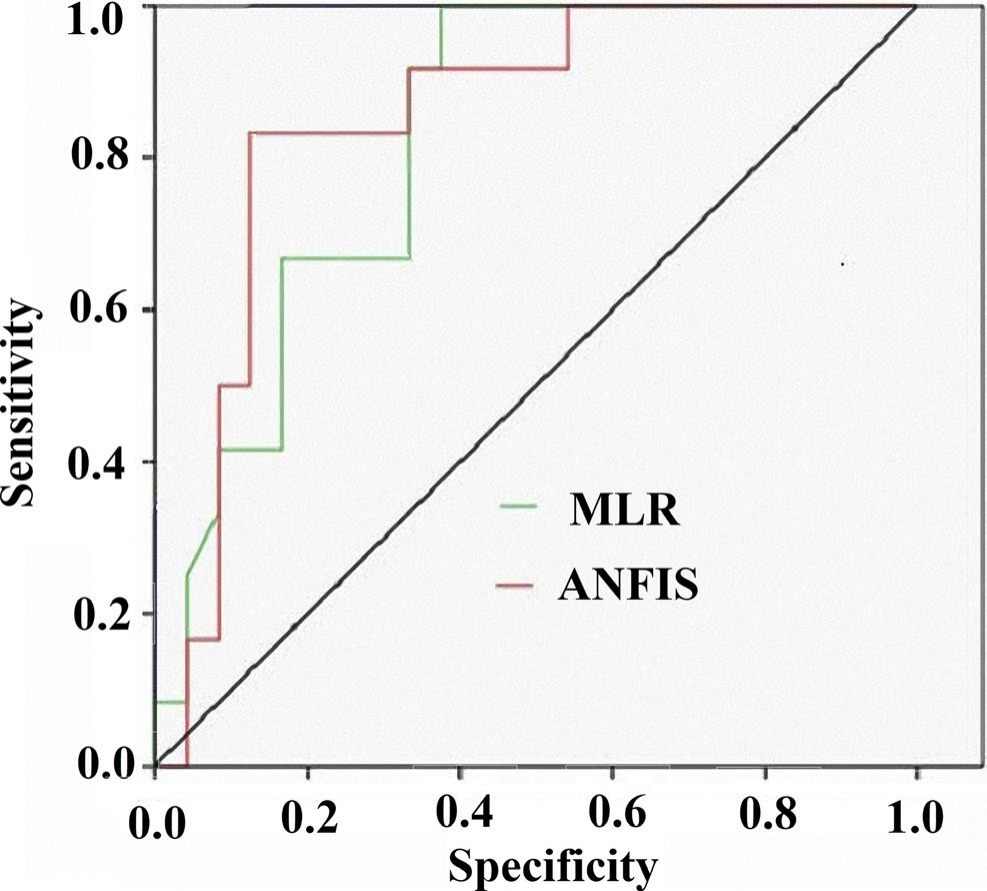
Success rate curves for the orange taste using MLR and ANFIS methods

ROC‐AUC analysis was used to evaluate the accuracy of the results. The 15 oranges and the associated values for the contributing factors were used for verification. The ROC curve showed that ANFIS and MLR had AUC values of 0.919 and 0.828. It demonstrates that ANFIS produced excellent to very good results (Figure 8 and Table 7).

**Table 7:** Area under the curve of built models

Models	Area	*SE*	Asymptotic significant	Asymptotic 95% confidence interval	
				Lower bound	Upper bound
MLR	0.828	0.087	0.002	0.697	0.920
ANFIS	0.919	0.067	0.001	0.797	0.980

**Figure 9:**
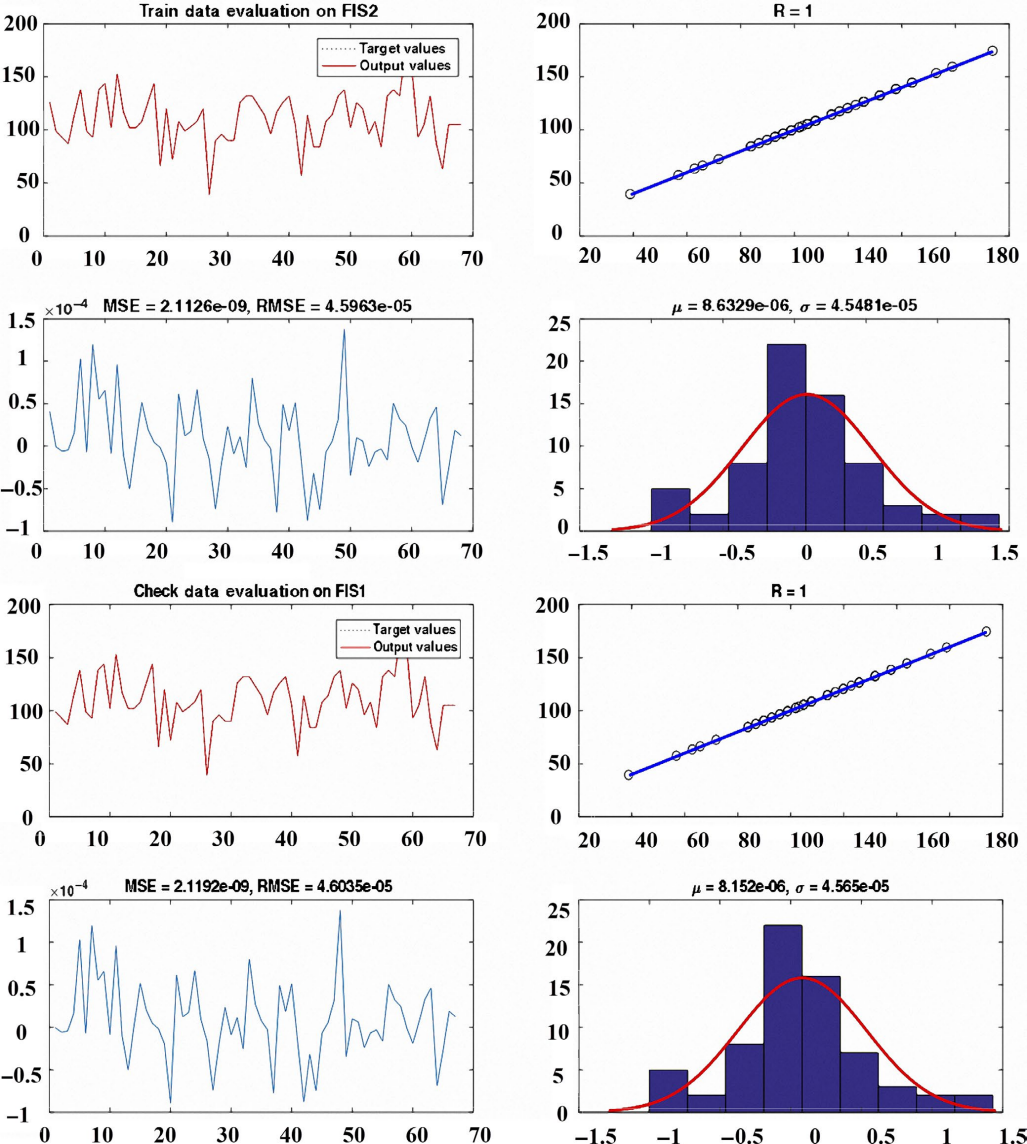
Accuracy of check and train data for prediction of orange taste

Also, in order to determine accuracy of modeling of orange taste, using ANFIS model were train and check data the output of modeling by ANFIS model and target value. The results showed that the model with low error for train and check data respectively had most accuracy for prediction of orange taste (Figure 9).

The input data and output data were fed into the ANFIS model to extract the rules. In fact, the ANFIS method is suitable where there is not enough information or extraction of rules is hard. Similarly Adelkhani et al, (2013)used ANFIS model to predict fruit quality. The results of them showed that the ANFIS method was suitable to predict fruit quality.

## 4 CONCLUSION

In this research, an attempt was made to predict the taste of orange in Fars Province, Iran, based on the MLR and ANFIS. This research developed a fuzzy logic model using the Sugeno fuzzy inference system. In the model to predict orange taste, vitamin C, acid, weight, fruit diameter, skin diameter, red, green, and blue values were used as input data. The rules were determined using ANFIS model in MATLAB software automatically. The ANFIS model according to train data and considering the lowest error defines rules. Moreover, the results show that the model with low error for train and check data respectively had most accuracy for prediction of orange taste. The advantage of this model compared to the other models was definition membership function according to train data automatically. In fact, definition membership function using ANFIS model and due to the reduction expert opinion causes the error probability to be zero.

## CONFLICT OF INTEREST

The authors declare that they have no competing interests.

## ETHICAL APPROVAL

Not applicable.

## CONSENT FOR PUBLICATION

Not applicable.

## INFORMED CONSENT

Written informed consent was obtained from all study participants.

## Data Availability

The datasets used and analyzed during the current study are available from the corresponding author on reasonable request.
